# Continuation-like semantics for modeling structural process anomalies

**DOI:** 10.1186/2041-1480-3-S2-S8

**Published:** 2012-09-21

**Authors:** Niels Grewe

**Affiliations:** 1 Institute of Philosophy, University of Rostock, Rostock, 18051, Germany

## Abstract

**Background:**

Biomedical ontologies usually encode knowledge that applies always or at least most of the time, that is in normal circumstances. But for some applications like phenotype ontologies it is becoming increasingly important to represent information about aberrations from a norm. These aberrations may be modifications of physiological structures, but also modifications of biological processes.

**Methods:**

To facilitate precise definitions of process-related phenotypes, such as *delayed eruption of the primary teeth *or *disrupted ocular pursuit movements*, I introduce a modeling approach that draws inspiration from the use of continuations in the analysis of programming languages and apply a similar idea to ontological modeling. This approach characterises processes by describing their outcome up to a certain point and the way they will continue in the canonical case. Definitions of process types are then given in terms of their continuations and anomalous phenotypes are defined by their differences to the canonical definitions.

**Results:**

The resulting model is capable of accurately representing structural process anomalies. It allows distinguishing between different anomaly kinds (delays, interruptions), gives identity criteria for interrupted processes, and explains why normal and anomalous process instances can be subsumed under a common type, thus establishing the connection between canonical and anomalous process-related phenotypes.

**Conclusion:**

This paper shows how to to give semantically rich definitions of process-related phenotypes. These allow to expand the application areas of phenotype ontologies beyond literature annotation and establishment of genotype-phenotype associations to the detection of anomalies in suitably encoded datasets.

## Background

The portion of reality under scrutiny by biology and medicine is much more exposed to the phenomenon of variability than, for example, chemistry or physics. Consequently, many biological truths only hold "normally" or "for the most part." If biomedical ontologies are considered to be information artifacts modeling or representing some portion of the underlying reality, they usually strive to capture only the aspects that are subject to some regularity because it seems that little knowledge can be gleaned from random aberrations.

In some areas, however, systematic considerations of the deviations from the normal case are of indisputable importance. One example for this is medical diagnostics, where pathological (and hence aberrant) phenotypes are a primary means for making inferences about the cause of a patient's condition. Ontologies that provide structured access to information about phenotypes are thus becoming valuable tools for researchers and clinical practitioners.

Examples of such ontologies include the Mammalian Phenotype Ontology [1] or the Human Phenotype Ontology [2]; both make use of the *Phenotype, Attribute and Trait Ontology *(PATO), which seems to have emerged as an accepted standard for specifying information about phenotypes [3].

The problem of the relationship between clinically normal and pathological is by itself troubling enough for the formally minded ontology engineer, and has, for example, driven research into the use of nonmonotonic logics (e.g. default logic) for this kind of application [4]. But it should also be noted that the problems arising from the distinction are further aggravated by the fact that the term "phenotype" is everything but a mono-categorial term. Phenotypes can describe not only traits pertaining to the concrete bodily structures, but also those which describe locations of such structures, dispositions or processes (cf. Table 1). Abnormal phenomena in each of these categories seem to deserve separate treatment; something that is neatly reflected by the fact that PATO defines the classes *process quality *and *physical object quality *as disjoint from one another; but the disjointness does not imply that both categories are completely unrelated: One will, for example, always assume that a quality of a process has something to do with the continuants participating in that process. For example, the process quality *rate of osmosis *of an osmosis process will, among other things, depend on the concentration of molecules in solution and the permeability of the membrane for the molecules in question.

**Table 1:** Phenotypes relating to different ontological categories

HPO ID	Phenotype	Related Category
HP:0010442	*Polydactyly*	Material Object
HP:0001100	*Heterochromia iridis*	Quality
HP:0008522	*High-frequency deafness*	Disposition
HP:0001696	*Situs inversus*	Location
HP:0000823	*Delayed puberty*	Process

Table illustrating that the term phenotype is used to describe traits in different ontological categories.

This suggests that it might be desirable to spell out process related phenotypes in terms of qualities of continuants. This issue should be separated from issues of causal or natural law like explanations of processes: A patient's tachycardia, for example, could be explained by an elevated level of norepinephrine in that patient's blood; but this is a causal explanation that could be part of a physician's diagnosis, not an explanation of what it means of a process to be a tachycardia, e.g. a certain state of the heart and the nervous system.

Such definitions are conspicuously absent from the *process quality *subtree of PATO, but its members are extensively used, for example, in definitions of the HPO. One example is the process quality *delayed*, which features in the definition of 47 classes in HPO, whether informally or explicitly referencing the PATO class PATO:0000502 (e.g. *delayed eruption of primary teeth*, HP:0000680). While this only accounts for less than half a percent of all HPO classes, it is an example of a certain type of process anomaly that could be termed a structural anomaly because it only affects the temporal order and contiguity of the process. This kind of anomaly does not seem to be very much involved in concrete biological problems that usually need to be considered for "material" anomalies of processes that arise from specific features of their participants or the relations between them (such as *decreased sensitivity of a process to oxygen*, PATO:0001554). Structural anomalies thus seem to be a useful subject for an initial case study of how anomalies of processes could be treated.

## Methods

In order to get a better picture of how to model structural anomalies of processes I will first elucidate what features accurately characterise the anomalies of processes. It will be useful to attempt this by considering analogues in continuants as a starting point. The reason for this is twofold: Firstly, as Edgar Dijkstra put it, "our intellectual powers are rather geared to master static relations and [...] our powers to visualize processes evolving in time are relatively poorly developed" [5]. We are thus less likely to run afoul of confounding intuitions by considering the "static" case of continuants first. Secondly, by differentiating structural anomalies of continuants from those of occurrents, we can expect to highlight the peculiarities of the occurrent variety that need to be taken into account. Only after these clarifications, a modeling framework that allows to accurately represent these features can be presented.

### Differences to continuant discontinuities

One continuant analogue readily presents itself if one considers some of the more serious siblings of delays, namely interruptions or disruptions (PATO:0001507) of processes. (I will use the terms "interruption" and "disruption" interchangeably.) If we consider instances of this kind of entity, we might speculate that there is some likeness between them and holes in continuants. The reason for this might be that we observe that every hole in a continuant corresponds to a discontinuity in the surrounding material [6], just as interruptions always coincide with discontinuities of processes.

But the analogy is imperfect at best for several reasons: (1) The surroundings of holes are mostly continuous, so that we can without any hesitation distinguish a hole in a piece of cheese from a gap between two distinct pieces of cheese. But since we specify processes as extending along a single temporal dimension, this distinction is no longer easy to make. Unless we want the difference between a gap and a "hole" to be blurred, this argument suggests that we need an identity criterion for processes and events that does not depend on temporal continuity.

(2) There seems to be no room for gradations of hole intensity: but clearly, a delay and an interruption in a process are interfering with the process in a similar way but with a different severity.

(3) Whether there are holes in a continuant is not at all affected by whether we think that it is normal or essential for the thing to have holes. This is not the case with interruptions and delays. For an episode within an event to be classified as an interruption or a delay requires that we also consider the normal or canonical course of the event.

For example, if Mary gets on a train in Berlin and off the train in Brussels, one cannot say that her travels have been interrupted simpliciter. We rather need to know whether she was traveling from Berlin to Brussels (no interruption) or from Berlin to London. In this case, it could be an interruption, but only if the normal course of events would not have involved a stop in Brussels. It might also be that something counts as an interruption on one level of description, but not on another. For example, Mary's train ride may be interrupted in Brussels even though her journey is not, e.g. if she decides to rent a car in Brussels to continue the journey.

I thus identify three points where process anomalies differ to a great extent from discontinuities of continuants. These need to be considered carefully when deciding how to model those anomalies:

1. We need identity criteria to re-identify events that contain interruptions.

2. We need to account for the differences between different kinds of structural anomalies (at least for delays and interruptions).

3. We need to establish a relationship between normal and anomalous tokens of a process type.

### Kinds of structural process anomalies

My proposal to tackle 1 and 3 will follow quite straightforwardly from the formal treatment of the matter, but 2 deserves some additional clarification. Firstly, there is an ambiguity about the meaning of "delayed". The corresponding process quality *delayed *is defined in PATO as follows:

A duration quality of a process inhering in a bearer by virtue of the bearer's duration which starts later than the natural start time. *(PATO:0000502)*

It seems that this definition does not encompass everything that would be called a delay. For example, Mary might be entitled to the claim that her travels from Berlin to London were delayed even if the delay did not result from the first train leaving later than it should have (with respect to the timetable) but rather from some unforeseen stop in Brussels. This concern is further amplified by the realisation that the phrase "natural start time" (of the process, that is) needs to involve some reference to an overarching process regarding to which the process in question is said to be delayed. For example, *delayed eruption of the primary teeth *might mean something different if one regards as the frame of reference the normal developmental process of a mouse or of a human being.

Secondly, our intuitions about the duration of a process are highly dependent on the severity of the process anomaly. While one usually would affirm that a process is still in effect during an episode that might be labelled a delay (and hence the delay contributes to the overall duration of the process), one would be hesitant to state the same thing about a disruption of a process: When there is a disruption of a process, we usually claim that the process is not taking place and hence the disruption episode should neither count as a part of the process in question nor should it contribute to the overall duration of that process. I will thus assume that the difference between delays and interruptions is due to different degrees of severity and affects how we determine the duration of the process. This is not to say that interruptions and delays cannot co-occur: An interruption of a subprocess might be closely correlated with a delay of its superprocess. For example, if Mary's train ride is interrupted a few times, she is effectively not riding the train during those interruptions. Still, the total duration of her journey increases through these interruptions because they can count as delays of the journey.

### Continuations in computer science

My approach to modeling structural anomalies of processes relies on the concept of continuations, which has been successfully employed by computer scientists to tackle a variety of seemingly divergent problems in the realm of programming language design and program analysis. A historical outline of the research on continuations, which also highlights their diverse areas of application, can be found in [7].

Roughly speaking, a continuation is an abstract data structure that represents a certain point in the control flow of a program by specifying the state of the computation at that point and how the computation will continue. A continuation thus specifies the "(meaning of the) 'rest of the program'" [8]. It is convenient to approach the topic of continuations by giving an example of their use. One such use is the transformation of a computer program written in an imperative language into a notation that can be interpreted in a functional way - something that is very useful when specifying the denotational semantics of a program.

Let us consider a common control flow operation in imperative programming languages: Returning control from a subroutine to the caller of that subroutine. For example, a function called *square*(*a*) in a computer program might compute the square of *a *and then return the computed value to the caller, which in turn might do additional computations with the obtained value, for instance compute its factorial (*fact*(*a*)), before yet again returning the result to its caller. With continuations, the control flow statement "*return*", required for returning control (and values) to the caller, can be disposed of. Instead, each function or subroutine can be written as taking an additional argument, namely the function which should be called with the result of the computation as an argument. That function is then the continuation of the subroutine in question because it specifies how the computation will continue. For instance, if we were computing the factorial of a square, we would write: *square*(*a*, *λs_a_. fact*(*s_a_*, *k*)), where the lambda term "*λs_a_. fact*(*s_a_*, *k*)" specifies what to do with the square of *a*, while *k *specifies what should be done with the result of computing the factorial. This kind of program formulation is aptly called "continuation passing style" [9].

For our present purpose continuations will show their usefulness if we do not consider their ephemeral variants which are merely applicable at a given point in the execution of the program, but rather continuations as "first-class" entities. This type of continuation allows the present execution state of the program to be stored alongside the information about how the execution is going to proceed. Such continuations are powerful enough to serve as models for various design patterns such as cooperative multitasking (coroutines), or exception and interrupt handling.

In the latter case some external intervention requires that the normal execution is suspended in order to take some immediate actions. With continuations, this can be conceptualized as saving the present continuation of the normal execution process and passing it to the subroutine that handles the interrupt, which will call it as its continuation after performing the necessary tasks.

### Process continuations

#### Preliminaries

These characteristics of continuations are useful when it comes to the structural anomalies of processes that I am considering here. My strategy will thus be to describe processes by associating them with their corresponding continuations such that for every point of time (except for the last) at which the process is in effect there exists a continuation of the process. This continuation describes the present state of the process and how it will continue. For example, consider the process of human childbirth, which might be divided into three phases: dilation, fetal expulsion and placental expulsion. Then the continuation of the first phase might refer to the state of the cervix being completely dilated and the fetus' head being positioned below the ischial spines, with the increased uterine contractions of the expulsion phase being the next subprocesses.

Since continuations in the realm of functional programming are purely mathematical concepts, they are devoid of any relation to time and just implicitly specify the required order of computation. This is an important difference to their intended use in the realm of process modeling.

Consequently, the way this proposal needs to be spelled out is highly dependent on the underlying ontology of time. But whereas all major top level ontologies (e.g. BFO, DOLCE or GFO) provide at least some account of time, it seems that a commonly accepted, standard ontological account of temporal phenomena has yet to emerge. Hence I restrict myself to explaining some of the prerequisites of my approach, all of which should be achievable no matter what top level ontology one chooses:

Since process continuations need to capture the present state of the process, the underlying ontology needs to contain complex ontological entities to model such states, e.g. through states of affairs [10], which represent the fact of something's being such-and-such; for example, a tomato's being red is a state of affairs composed of the tomato and the quality *red *inhering in that tomato.

Also, modelling anomalies requires insight into the internal structure of processes. It thus needs to be possible for processes to be made up from subprocesses. Hence I will assume that processes can, but need not, have temporal parts.

Furthermore, since processes usually involve things changing, each process needs to be associated with (at least) an initial or input state and a final or output state [11]. In a weaker sense, a process might also be an episode of absence of change. In this case, the initial and final state are identical and there will be a continuation for every minimally extended period of rest.

Above all this, I will assume that the underlying formalisation of time is such that two processes in direct succession coincide at a common boundary, something that is made explicit in the BFO top level ontology by the class *ProcessBoundary *[12]. This way, it is possible to claim that the final state of the first process might serve as the initial state of the second process. With regard to the first process, the boundary will be called a right boundary, with regard to the second process the boundary will be called a left boundary. This requirement is sufficient to express "conventional" change, where the separation of an event into subevents is such that the result of the preceding event is "picked up" by the succeeding event (i.e. wherever succeeding steps can be identified). Hence, the requirement might not be sufficient to express continuous change or so called "Cambridge change" [13], where the change occurs between two contrary or contradicting states. To handle this kind of change, more complex formal machinery, such as the theory of boundaries sketched in the GFO [14], might be needed. Adapting the modeling strategy presented here should be easily possible.

#### Anomaly-invariant process descriptions

With these provisos, I will first attempt do give a general framework for describing processes in a way that is neutral to structural anomalies. The initial building block of this framework is the definition of what it means to be a continuation of an event or process. And although I have up to now used the terms "event" and "process" interchangeably because a principled distinction between them is outside the scope of my present endeavour, I will in these definitions usually refer to the occurrent entity as an event, which is in line with terminology from Galton and Mizoguchi [11], who reserve the term "event" for occurrents that can be regarded as complete wholes, whereas "process" describes an occurrent with almost continuant like characteristics that is the "stuff" that events are made of. For example, the incision event of an apendectomy would be said to be "made of" a cutting process. That being said, the continuation of an event can be defined as follows:

**Definition 1: ***κ *is a continuation of the event *e *iff

1. *κ *is a continuant.

2. for every timepoint *t *and every independent continuant *c*, if *e *is in effect at *t *and *κ *exists at *t *and *e *is ontologically dependent on *c *at *t*, then *κ *is ontologically dependent on *c*.

3. there exists some proper subevent *e_c _*of *e *and a timepoint *t*, such that the right boundary of *e_c _*is at *t *and the left boundary of *κ*'s life-time is also at *t*.

4. there exists some proper subevent *e_s _*of *e *and some state of affairs *s_c _*such that

(a) *s_c _*is the final state of *e_c _*and *κ *is ontologically dependent on *s_c_*.

(b) the left boundary of *e_s _*coincides with the right boundary of *κ*'s life-time and *s_c _*is the initial state of *e_s_*.

In this definition, clause 1 is more than just a play on words. Continuations also have to be (dependent) continuants because they fulfill the canonical definition of a continuant as a thing that is wholly present at every point of its existence. The reason for this is that we want to assume that the continuation comes to be once all the conditions relevant for advancing the course of events obtain.

A crucial part of these conditions is specified in clause 2: If the process is ontologically dependent on some entity at a given stage (meaning that the entity participates in the process), the continuation cannot exist without that entity's continued existence. The dependence relation might be a generic one, though. For example, a game of chess depends on a certain set of chess pieces at every stage of the game. But for the game to continue, it is not necessary that the pieces involved remain numerically identical. I can very well continue playing the game if I replace one white pawn with a different one, provided that I place it in the correct position.

With clause 3, the definition stipulates that a continuation has to be the current continuation of at least one subevent of the overarching event *e*, namely of the subevent up to which the event has successfully progressed. This requirement is closely related to clause 4a. This clause specifies that the continuation depends on the state of affairs that is the final state of the subevent of which the continuation is the current continuation. I will call this state of affairs the *context state *of *κ*. Conversely, by clause 4b, that state must also be the initial state of the succeeding subevent, so that the continuation really specifies how the event will continue.

Continuations thus are not themselves parts of the event, but serve as "interfaces" between its different subevents. This, to my mind, suggests that the distinction between an event and its continuations is orthogonal to the process/event distinction made by Galton and Mizoguchi, where the event is "composed of" its constituent processes [11].

We can easily apply this definition to the example of childbirth: consider the timepoint *t *at which the cervix is fully dilated. At this point in time, the event clearly depends on both mother and child. Hence, a continuation at *t *will also depend on these entities (clause 2). Furthermore, there is a subevent of the childbirth event that ends at *t*, namely the dilation of the cervix (clause 3). Since that event has a final state that is a complex of the fetal station deep inside the maternal pelvis (but still inside the uterus) and the cervix' being dilated, the continuation has that state as its context state (clause 4a). Finally, when childbirth continues (at *t*) with its next subevent (the fetal expulsion phase), the context state (which is also the input of this subevent) no longer obtains due to the fetus moving further down the pelvis and the continuation thus also disappears again at *t *(clause 4b), allowing the remainder of the event to unfold. But at any rate, the definition allows for a great deal of variability. It does not, for example, stipulate that the subevents related by the continuation are contiguous, something that is crucial for the purpose of modeling interruptions. Still all crucial information about the event is represented in its continuations. It is hence useful to define the *continuation set *of all continuations of *e *as well:

**Definition 2: **Let *e *be an event, then *K_e _*is the continuation set of *e *iff

1. for every continuation *κ*, if *κ *is a continuation of *e*, *κ *∈ *K_e_*.

2. for every proper subevent *e_s _*of *e*, if *K_s _*is the continuation set of *e_s_*, then for every *κ_s _*∈ *K_s_*, *κ_s _*∈ *K_e_*.

The second clause is expendable if transitivity of the subevent relation is assumed. From the vantage point of classical mereology, this assumption is quite plausible, but there may be some rationale for dropping it in the case of processes [15]. For example, one might wish to claim that depressing the accelerator pedal is a subevent of driving a car, and that moving the foot down is a subevent of pushing the accelerator pedal, but that moving the foot down is not a subevent of driving a car - obeying the intuition that depressing an accelerator pedal is in a strong sense "part" of driving a car, while foot movement is not. I do not, however, hold any strong opinions on the matter. But even if one adopts such a view, it should be possible to claim that there can be interruptions or delays during episodes that are not subevents in a restricted sense. With this kind of arcane subevent relation, the continuation set of *e *will contain more than just continuations of *e*. The definition of continuation sets is thus neutral with regard to this kind of ontological decision. In the childbirth example, the continuation set would consist of the continuations that describe the beginning of the event and the transitions from the dilation phase to the fetal expulsion phase and from there to the expulsion of the placenta, along with all continuations of these processes (esp. contractions of various degrees).

But the continuation set alone is not enough to capture a process in its entirety because it is easy to observe that for the very end of the event there cannot be a continuation (clause 4b of definition 1 would be violated). One thus has to take into account the final state of the entire event:

**Definition 3: **Let *e *be an event, *K_e _*the continuation set of *e*, and *s *the final state of *e*. Then 〈*K_e_*, *s*〉 is the event description of *e*.

For childbirth, the final state needed in addition to the continuation set mentioned above obviously is "being located outside the maternal body" for both child and placenta.

The notion of an event description for individual events can then be used to formulate class-level definitions of event types, by specifying *continuation signatures *that characterise types of events:

**Definition 4: **〈*κ*, *S*〉 is a *continuation signature *iff

1. *κ *is an ordered set of continuation types.

2. *S *is a state type.

3. there exists some *s*, *κ*_1_,. . . *κ_n _*such that

(a) *s *is an instance of *S*.

(b) the instantiation relation maps *κ*_1_,. . . *κ_n _*to exactly one element of Σ.

(c) 〈{ *κ*_1_, *... κ_n_*}, *s*〉 is the event description of some event.

This is basically a class-level reinterpretation of an event description that ensures that an event instantiating the signature actually exists. The instantiation relation between event tokens and their types is then defined in terms of instantiation of continuation signatures:

**Definition 5: **Let *e *be an event, *E *an event type and 〈Σ, *S*〉 the continuation signature of *E*. *e *is an instance of *E *iff there exists an event description 〈*K_e_*, *s*〉 of *e *such that

1. *s *is an instance of *S*.

2. for every *κ *∈ *K_e_*, *κ *is an instance of some element of Σ.

3. for every type *T *∈ Σ there is an instance of *T *in *K_e_*.

In this view, event types are distinguished not only by what their instances bring about but also by how they bring it about. They are thus strictly linear; variance in events, as is caused by conditional or alternative subevents, would thus require additional aggregation of event types.

These definitions provide the basic framework for describing processes in a way that is invariant to anomalies, so that we can now give an accurate account of the different types of anomalies.

## Results and discussion

### Anomaly kinds and anomaly invariance

I will now show that it is possible to give compelling definitions of delays and interruptions using the framework sketched above. In the course of this, I will also show that event descriptions using continuation signatures are in fact invariant with respect to these anomaly kinds. This can be achieved by showing that both normal and anomalous tokens of what intuitively seems to be the same type of event actually belong to the same event class because the event description (definition 3) of each instantiates a common continuation signature (definition 4).

#### Delays

I will start with presenting the definition of a delay:

**Definition 6: **Let *e *be an event of type *E *and *K_e _*the continuation set of *e*. The proper subevent *e_d _*of *e *is a delay of *e *iff

1. *e_d _*is a proper subevent of a delay of *e*.

2. or

(a) *e_d _*is temporally contiguous.

(b) there exists *κ *∈ *K_e _*such that *κ*'s life-time is longer than *e_d_*.

(c) the right boundaries of *e_d _*and of *κ*'s life-time coincide.

This definition does justice to the intuition that delays contribute to the overall duration of a process. The episode *e_d _*is part of the overarching event, but it does not contribute anything to advancing the normal course of events because the continued existence of the continuation for the next genuine subevent of the process requires that all participants and the final state of the previous genuine subevent also continue to exist, and hence no changes relevant to the process can occur. Readers should note that in PATO the process quality *delayed *is not attached to the superprocess that experiences the delay, but the subprocess immediately succeeding the delay. Also, if we contrast an event *e *which contains a delay *e_d _*with *e' *which is identical to *e *except for not containing *e_d_*, we see that *e *and *e' *contain exactly the same continuations in their respective continuation sets. The reason for this is that the continuation *κ *in existence during *e_d _*is not a "new" continuation but instead one that existed before the delay came about (and is hence shared with *e'*). Both delayed and normal events thus trivially instantiate the event class *E*.

In the childbirth example, suppose that after complete dilation of the cervix there is an episode during which the mother does not experience further contractions. During that episode, the context state (cervix dilated; fetus inside the uterus; fetal head engaged in the pelvis) still obtains and all the participants are present. Consequently, the continuation continues to exist until the uterine contractions resume. It stops existing together with the episode, which hence counts as a delay of the childbirth.

#### Interruptions

Interruptions can be defined similarly but with additional effort:

**Definition 7: **Let *e *be an event of class *E *and *K_e _*the continuation set of *e*. The event *e_i _*is an interruption of *e *iff

1. *e_i _*is a proper subevent of an interruption of *e*.

2. or

(a) *e_i _*is temporally contiguous.

(b) the left and right boundaries of *e_i _*lie between the left and right boundaries of *e*.

(c) the temporal extensions of *e *and *e_i _*do not overlap.

(d) there exists a continuation *κ *and a state of affairs *s*, such that

i. *κ *∈ *K_e_*

ii. *s *is the final state of *e_i_*.

iii. *s *is the context state of *κ*.

iv *κ *existed at or before the left boundary of *e_i_*.

v. a left boundary of an episode of *κ*'s life-time coincides with the right boundary of *e_i_*.

The complexity in this definition is due to the fact that it needs to account for the intuition that interruptions do not contribute to the overall duration of the process. It basically assumes that an interruption is something that fills a "gap" in the process. Interruptions further differ from delays in that the necessary prerequisites for continuing the process are not present during the interruption.

Consequently, a continuation cannot be present during the interruption. The continuation that characterises the process' course up to the interruption is rather present sometime before the interruption (most likely at its left boundary) and it reappears once the prerequisites for continuing the process have been reestablished. Continuations can thus be intermittently existent, which makes them a bit awkward, but not any more awkward than ordinary objects that exist only intermittently [16], for example a table that is disassembled before it is moved to another room where it is reassembled. Likewise, the continuation will be the same continuation when it "reappears" and no change to the continuation set needs to be made in order to accommodate interruptions. And since the identity of the event depends on its continuations, the same event is present before and after the interruption. Furthermore, the same argument as with delays reveals that the continuation signature of the event type will also stay the same, thus allowing the interrupted event to be subsumed under the same event type as the event modulo interruption.

For an example, we cannot continue to entertain a high-level view of childbirth because it is rarely properly interrupted. Instead, let us consider the case of the uterine contractions throughout childbirth, which might be interrupted. For my purposes, I will consider a contraction to consist of a contraction phase, a relaxation phase and a latency phase before the next contraction occurs. The continuations of the first two phases can be determined quite straightforwardly, but the latency phase, which does not consist of change but of rest, poses a little problem: How will we distinguish a delay succeeding the relaxation phase from the latency phase? This is possible by acknowledging that a period of rest can be interrupted or delayed not only at specific moments, but continuously at every possible moment during its occurrence. To model the latency phase, I thus need to introduce a number of continuations that continue minimally extended periods of rest. That way, the absence of change that is essential to the process can be distinguished from abnormal periods of such absence.

One possibility of what it means for the uterine contractions to be interrupted readily presents itself when one considers that the contractions depend on the presence of oxytocin [17]. A sufficient concentration of oxytocin in the maternal blood will thus be part of the context state for all continuations of uterine contractions under labour. If a drop in the oxytocin level caused a cessation of the contractions, the resulting "gaps" in the overall contraction process would need to count as interruptions (of the continued contraction process, but not of the overall childbirth!) because without presence of the context state, the continuation cannot exist either.

### Applications and limitations

Common use cases of phenotype ontologies, for example establishing genotype-phenotype mappings, do not require further analysis of anomalies. But such analysis might prove to be much more fruitful for other use cases, for example in applications that try to detect such anomalies in datasets. For example, given a description of the natural developmental process of a child, one could detect instances of *delayed eruption of primary teeth *(HP:0000680) from a set of instance data by selecting the tooth eruption subprocess and checking whether the preceding continuation exists longer than the process it is a continuation of. If so, the tooth eruption has been delayed.

Similarly, one could distinguish between interruptions and delays of chemical reactions. We would say that a reaction is delayed when the preceding reaction produced the necessary reactants for the following reactions but, for example, the enzyme catalysing the reaction is inhibited because of the pH or the concentration of other molecules. If on the other hand, one of the products of the preceding reaction would be removed from the system, we would be more inclined to categorise it as an interruption.

This example also shows the limits of the present approach. In the usual case of chemical reactions in biological contexts, the behaviour of collections of molecules is described in a statistical way, that is, not all molecules participate in a reaction and that reaction is usually part of a steady state system so that at every point in time different molecules participate in it. Ontological modeling and analysis of such systems requires a more sophisticated approach.

There are two other situations that cannot be modeled with this approach. For one, it is strictly not possible to arrive at a satisfactory understanding of abortions or missing process parts using continuation signatures. This is because they treat processes as *complete *processes by definition and the removal of subevents from these processes does not leave continuation sets unscathed. To me, this suggests that this kind of anomaly does not fall into the same category as interruptions and delays.

Additionally, some kinds of delays seem to lack the discrete nature that is required for the definitions presented here. Think, for example, of a growth process that is being delayed not because it is stalled at some stage for a specific period of time, but rather because is proceeds at reduced speed throughout its course. Here the delay is continuously accumulated while the proper process remains in effect.

## Conclusions

I have presented a modeling approach that tries to capture certain types of process anomalies which are characterised by only affecting the temporal structure and continuity of the processes. This scheme accurately represents the differences between anomalies of different strength, explains the unity of the parts of an interrupted process, and provides criteria for why anomalous and normal tokens can both belong to the same event or process class. It has been shown that using this approach a more detailed semantics for process related phenotypes can be given; though it remains dubious whether this is of great utility for conventional use cases of phenotype ontologies.

Furthermore, the concept of continuations that was used to obtain these results originated from a programming language design context, so that it also serves as a reminder that work from the more theoretical computer science community can be fruitfully applied to ontology engineering.

I have also uncovered a few blind spots that provide interesting avenues for future research; for instance with regard to aborted processes, continuous delays, or the modeling of processes affecting collectives. But apart from these things, there also remains the latent issue of proper treatment of all temporal phenomena in ontologies. I have tried to avoid this issue here by giving the general requirements of my approach with regard to temporal modeling. But still a sensible and generally agreeable scheme for dealing with time and occurrent entities remains a considerable desideratum for all ontology modeling.

## Competing interests

The author declares that he has no competing interests.

## References

[B1] Smith CL Goldsmith CAW Eppig JT 2The Mammalian Phenotype Ontology as a tool for annotating, analyzing and comparing phenotypic information Genome Biology 2005 6 R7 10.1186/gb-2005-6-5-p7 15642099 PMC549068

[B2] Robinson PN Kohler S Bauer S Seelow D Horn D Mundlos S The Human Phenotype Ontology: a tool for annotating and analyzing human hereditary disease American Journal of Human Genetics 2008 83 610 615 10.1016/j.ajhg.2008.09.017 18950739 PMC2668030

[B3] Mungall CJ Gkoutos GV Smith CL Haendel MA Lewis SE Ashburner M Integrating phenotype ontologies across multiple species Genome Biology 2010 11 R2 10.1186/gb-2010-11-1-r2 20064205 PMC2847714

[B4] Hoehndorf R Loebe F Kelso J Herre H Representing default knowledge in biomedical ontologies: application to the integration of anatomy and phenotype ontologies BMC Bioinformatics 2007 8 377 10.1186/1471-2105-8-377 17925014 PMC2180186

[B5] Dijkstra E Go To Statement Considered Harmful Communications of the ACM 1968 11 3 147 148 10.1145/362929.362947

[B6] Lewis DK Lewis S Holes Australasian Journal of Philosophy 1970 48 206 212 10.1080/00048407012341181

[B7] Reynolds JC The Discovery of Continuations Lisp and Symbolic Computation 1993 6 233 248 10.1007/BF01019459

[B8] Wadsworth CP Continuations Revisited Higher-Order and Symbolic Computation 2000 13 131 133 10.1023/A:1010074329461

[B9] Sussman GJ Steele GL Jr Scheme: A Interpreter for Extended Lambda Calculus Higher-Order and Symbolic Computation 1998 11 405 439 10.1023/A:1010035624696

[B10] Armstrong DM A World of States of Affairs 1997 Cambridge: Cambridge University Press

[B11] Galton A Mizoguchi R The water falls but the waterfall does not fall: New perspectives on objects, processes and events Applied Ontology 2009 4 71 107

[B12] Grenon P BFO in a Nutshell: A Bi-categorial Axiomatization for BFO and Comparison with DOLCE IFOMIS Report 06/03, University of Leipzig, Leipzig 2003

[B13] Mortensen C Zalta EN Change and Inconsistency The Stanford Encyclopedia of Philosophy 2011 http://plato.stanford.edu/archives/fall2011/entries/change/

[B14] Herre H Heller B Burek P Hoehndorf R Loebe F Michalek H General Formal Ontology (GFO). Part I: Basic Principles 2006 Onto-Med Report 8, University of Leipzig, Leipzig

[B15] Seibt J Free Process Theory: Towards a Typology of Occurrings Axiomathes 2004 14 23 55 10.1023/B:AXIO.0000006787.28366.d7

[B16] Simons P Parts. A Study in Ontology 1987 Oxford: Clarendon Press

[B17] Ferguson JKW A study of the motility of the intact uterus at term Surgical and Gynecological Obstetrics 1941 73 359 366

